# NN-VRCTST: Neural Network Potentials Meet Variable Reaction Coordinate Transition State Theory for the Rate Constant Determination of Barrierless Reactions

**DOI:** 10.1021/acs.jctc.5c01288

**Published:** 2025-10-09

**Authors:** Simone Vari, Carlo de Falco, Carlo Cavallotti

**Affiliations:** † Dipartimento di Chimica, Materiali e Ingegneria Chimica, Politecnico di Milano, 20131 Milano, Italy; ‡ MOX, Modeling and Scientific Computing, Dipartimento di Matematica, Politecnico di Milano, 20133 Milano, Italy

## Abstract

The determination of rate constants for barrierless reactions poses severe problems from a theoretical perspective. The main challenges concern the proper description of the electronic structure of the reacting system, which may have multireference character, the anharmonicity of the relative motions of the fragments, and the proper definition of the reaction coordinate. The literature state of the art in the context of transition state theory is its variable reaction coordinate implementation (VRC-TST), which overcomes these difficulties in determining the number of transition state ro-vibrational states through a Monte Carlo sampling of the potential energy surface (PES) defined by the relative orientation of the two fragments. Although approaching the accuracy of experiments, VRC-TST requires tens of thousands of single-point energy (SPE) evaluations, thus being computationally demanding. The approach developed in this work, named NN-VRCTST, aims at fitting the PES with physics-inspired artificial neural network (ANN) models to be used as surrogate potentials in VRC-TST simulations. The ANN efficacy is evaluated in the computation of high-pressure limit rate constants for gas-phase barrierless reactions and validated over state-of-the-art VRC-TST simulations. It is shown that the NN-VRCTST tool reaches an accuracy within 20% with respect to VRC-TST simulations performed by using traditional approaches. While lowering the number of SPE needed by at least a factor of 4, the computational framework devised here allows one to decouple ANN training and VRC-TST calculations, enabling the optimization of the SPE evaluations as well as the quality inspection of the employed data points. We believe that the NN-VRCTST approach has the potential to evolve into a robust and computationally efficient framework for performing VRC-TST calculations for barrierless reactions.

## Graphical abstract

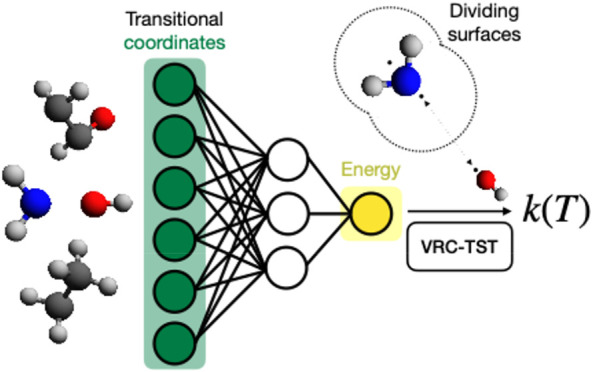

## 1 Introduction

The determination of rate constants for barrierless reactions remains a persistent challenge in chemical kinetics.^1–4^ These reactions are so named because they take place without passing a well-defined saddle point, resulting in the absence of a characteristic energy barrier on the potential energy surface (PES) along the minimum energy path (MEP) connecting reactants and products. The description of barrierless reactions enables the investigation of the pathways that follow the recombination of two radicals, which is of pivotal importance for astrochemistry, combustion, and atmospheric chemistry. The reasons why the determination of rate constants for barrierless reactions is theoretically challenging are mainly three: (i) the proper description of the electronic structure of the reacting system, which may have considerable multireference character. This is generally the case when both reacting fragments are radicals whose interaction leads to the formation or cleavage of covalent bonds. In this case the interaction energy should be evaluated using multireference electronic structure methods, which are traditionally computationally demanding and require expert supervision for accurate modeling of the MEP; (ii) the significant anharmonicity of the relative motions of the two reacting fragments, which are large-amplitude motions and therefore badly described in the rigid rotor harmonic oscillator (RRHO) approximation; and (iii) the proper definition of the reaction coordinate, which poses some challenges in the application of transition state theory (TST).

Different approaches have been proposed in the literature for the evaluation of the rate constants for barrierless reactions. Many are based on TST, either in its microcanonical or canonical forms, and on the variational minimization of the sum of states as a function of the reaction coordinate.^5,6^ An approach that has extensively been used in the literature is a form of variational TST (VTST) in which the density of states (DOS) is computed along the reaction coordinate in the RRHO approximation for all internal degrees of freedom, with the exception made for the internal motions that degenerate into translational and rotational motions, usually referred to as the transitional degrees of freedom. The DOS of these anharmonic motions can then be evaluated using suitable reduced dimensionality models, such as the hindered Gorin model.^7–9^ An alternative approach is the statistic adiabatic channel model (SACM), in which the sum of states at the TS is evaluated by the quantum resolved direct count of all product states.^10^ Both approaches have shortcomings, related to either the difficulty of unequivocally defining the Gorin hindrance angles or the complexity of applying SACM to large molecules. Another alternative, which is implemented in several master equation codes such as MESMER,^11^ MultiWell,^12^ as well as the RMG master equation solver,^13^ is to use inverse Laplace transformation (ILT) to determine the DOS at the transition state, and thus microcanonical rates. Although the ILT approach has been extensively used in recent years,^14^ it requires as inputs accurate rate constants for the recombination process in an ample temperature range, so that it can not be considered as a predictive model, but rather as a method to generate microcanonical rates to be used as input in master equation simulations. At present, variable reaction coordinate transition state theory (VRC-TST)^15–17^ is generally considered as the gold standard for modeling barrierless reactions. VRC-TST, which involves constructing complex dividing surfaces anchored on reactive centers to compute and integrate the reactive flux over transitional degrees of freedom, properly addresses the three challenges mentioned above for the estimation of barrierless rate constants provided that the PES is constructed by using multireference methods. VRC-TST has demonstrated remarkable accuracy for gas-phase reactions, often aligning closely with experimental results, so that errors as small as 20% are generally expected when the PES sampling is performed at the highest level of theory.^1,17–19^ This methodology is implemented in specialized software such as VaReCoF,^19^ PolyRate,^20^ and RotdPy,^21^ with automation accelerating the setup of the necessary inputs,^22^ facilitated by tools like EStokTP^23^ or AutoMech.^24^ Coupling EStokTP with VaReCoF allows precise rate constant calculations,^18,25–29^ aided by corrections to PES sampling to account for methodological simplifying assumptions used to accelerate the calculations, related for example to the use of reduced basis sets or active spaces, and geometry relaxation. A crucial step in the determination of the energy *E* and angular momentum *J* resolved rate constant obtained from VRC-TST is the calculation of the number of available states of the transition state, which involves an on-the-fly Monte Carlo sampling of the PES through extensive single-point energy (SPE) calculations, usually on the order of 10^4^, via electronic structure software. This computational demand poses a bottleneck, especially for larger reacting fragments. Figure 1 illustrates the substantial number of SPE calculations required for selected gas-phase barrierless reactions using VRC-TST.

**Figure 1:**
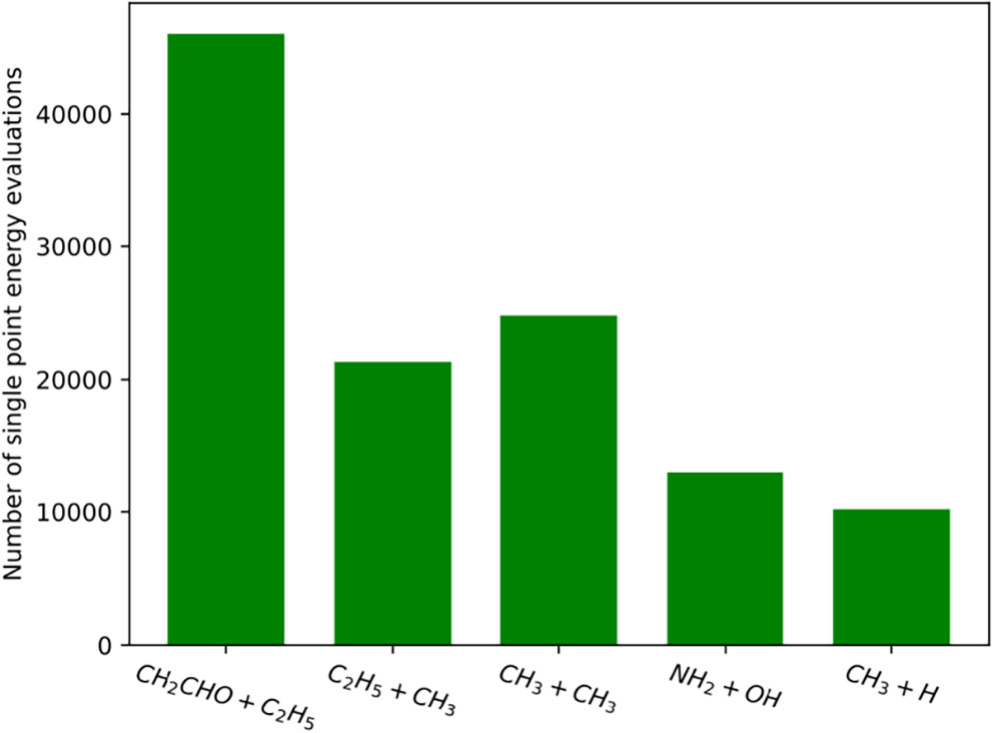
Number of single-point energies calculations performed with electronic structure software for a full VRC-TST simulation for a selected number of barrierless gas-phase reactions. Calculations were performed using an orientational integration convergence threshold of 5% for the flux and a maximum number of SPE evaluations of 5000 for each dividing surface, although generally a smaller sampling is needed to reach the convergence threshold. The minimum number of sampling points was 200.

Within this framework, Artificial Intelligence (AI) offers immense potential. Applications of AI in gas-phase chemical kinetics cover a broad range of topics. Much work has been devoted to the development of machine learning models for predicting molecular^30,31^ and thermodynamical^32^ properties as well as reactivity.^33^ Recent efforts put forward deep learning models for transition state optimization^34^ as well as for fitting ab initio PESs,^35–42^ an issue approached from different perspectives that led to the development of many specialized package suites,^43–46^ of which we provide just a selected number of references without the intent to make a comprehensive list. Some of the advantages that machine learning could bring to VRC-TST have already been explored:^47^ a Behler-Parrinello neural network^48^ as developed in the Amp package^49^ was implemented by Chen and Goldsmith, showing a significant reduction in the number of SPE effectively calculated. Additional advantages are given by the decoupling of the estimation of the SPE energies from the VaReCoF simulations, which allows a more efficient use of HPC resources as well as the possibility to perform preliminary tests of the convergence, in particular, for what concerns the active space, of the multireference simulations used to construct the PES. The aim of this work is to further investigate the synergy between VRC-TST and AI, addressing the reconstruction of the multidimensional PES by means of an artificial neural network (ANN) model. This ANN is specifically designed to incorporate relevant physical information into its architecture and to remain consistent with the theoretical framework of VRC-TST, respecting the frozen fragment geometries approximation. To achieve this, the ANN is parametrized using the transitional coordinates employed in VRC-TST integral evaluations, which serve as descriptors of the molecular configuration. This approach not only enhances the model physical interpretability but also enables future applications in frozen fragments dynamical simulations.^50^ Moreover, the ANN model is embedded into the consolidated workflow provided by EStokTP and VaReCoF through a direct interface, delivering rate constants without relying on external codes.

This article is organized as follows. In Section 2, the methods developed and applied are discussed, and particular emphasis is devoted to the explanation of VRC-TST and the ANN model, as well as to the data set production phase. This is followed by the results and discussion section that shows the performances of our model in terms of high-pressure rate constants prediction for some selected systems. The last section concerns the conclusion and the future developments of this work.

## 2 Methods

### 2.1 VRC-TST Calculations with ANN Potential

The most used and successful implementation of VRC-TST consists in the application of variational TST to variable, multifaceted transition state dividing surfaces, defined by the positions and distances of pivot points, and in the estimation of the number of available states (NOS) over the PES determined by the relative motions of the two reactive species over the dividing surface.^16,17^ The two reactive species are treated as rigid fragments, thus limiting the degrees of freedom (DOF) to a maximum of six and avoiding the description of intramolecular motions, which are therefore assumed to be spectators to the reactive process. For interactions involving a nonlinear and a linear fragment, the DOF reduces to five, and for a nonlinear fragment interacting with an atom, to three. The set of gas-phase reference systems selected to benchmark this approach against theoretical predictions comprises the following reactions: CH_3_ + CH_3_, C_2_H_5_ + CH_3_, NH_2_ + OH, CH_3_ + H, CH_2_CHO + C_2_H_5_. Consequently, the potential energy surface explored during Monte Carlo sampling becomes a multidimensional scalar function spanning three to six dimensions. These DOF values correspond to transitional coordinates, which are associated with large-amplitude motions. Figure 2 illustrates these transitional coordinates, comprising one distance (RTS), two angles (AABS1, AABS2), and three dihedrals (BABS1, BABS2, and BABS3). In the traditional VRC-TST implementation, the PES is evaluated on the fly during the Monte Carlo sampling of the NOS of each dividing surface. When the barrierless reaction involves the recombination between two radicals, as it is the case for the reactions considered in this work, the PES sampling is properly performed using a multireference level of theory, usually CASPT2, with a small active space and basis set, even if other valid alternatives emerged recently in the literature.^51^ At the lowest level of theory, often a (2e,2o) active space, composed of electrons in the bonding and antibonding orbitals of the bond being broken (or formed) in the investigated reaction and the cc-pVDZ basis set are sufficient to obtain relatively accurate rate constant estimations (within a factor of 2–3 from experiments or higher-level calculations), as long as a correction potential is used to improve the accuracy of the calculated energies. This is, for example, the case for recombination reactions between alkyl radicals.^17^ The selected active spaces for the reference systems are CH_3_ + CH_3_ (2e,2o), C_2_H_5_ + CH_3_ (2e,2o), NH_2_ + OH (6e,4o), CH_3_ + H (2e,2o), and CH_2_CHO + C_2_H_5_ (4e,4o). The (6e,4o) active space of NH_2_ + OH is formed by the (3e,2o) p orbitals and electrons of oxygen not involved in the O–H bond for hydroxyl and by the radical electron and orbital and the N lone pair of the amino radical (3e,2o), while the (4e,4o) active space for CH_2_CHO + C_2_H_5_ is formed by the electrons and orbitals of the two radical centers (2e,2o) and by the bonding and antibonding π orbital of the vinoxy radical (2e,2o). A level shift of 0.2 is employed in all CASPT2 calculations to mitigate intruder states effects. It is here important to notice that avoiding errors in CASPT2 energy estimates due to intruder states, as well as proper selection of the active space, are necessary requisites in order to perform accurate VRC-TST calculations. Developing a successful CASPT2 computational strategy can be complicated and requires considerable experience. This is the reason why alternative robust computational approaches tailored for VRC-TST energy estimates have been recently proposed by Crisci et al.^22,51^

**Figure 2:**
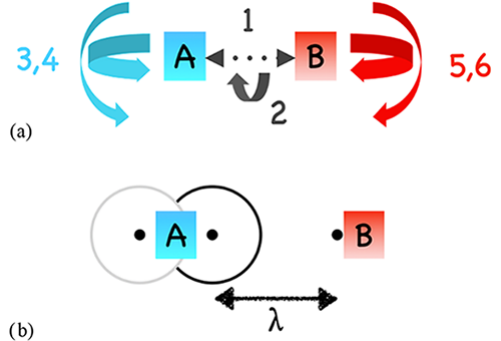
(a) Six large-amplitude motions, also called transitional motions, associated with two nonlinear fragments A and B are shown as transitional coordinates between two frozen fragments; in (b), the dots represent pivot points placed close to the reactive centers. Lambda is the distance between two pivot points placed on two different fragments.

In the present work, the sampling step is realized replacing each call to an electronic structure software by a call to an ANN model, which receives as input the transitional DOF values and returns the PES value. The energies so determined are corrected using a one-dimensional function of RTS, the correction potential, which can thus be estimated through a limited set of accurate calculations.

The correction potential comprises two contributions, the first for the geometry relaxation and the second for the level of theory (high-level correction). The former is computed as the difference between the energy of the relaxed geometry of the MEP, determined in the present work at the uωB97X-D/jun-cc-pVTZ level of theory, and the energy of the geometry obtained relaxing only the transitional degrees of freedom at each given distance between 2 and 4 Å, keeping the geometry of the fragments frozen. As it is a correction term for a relative change between a slightly distorted and a relaxed geometry, it is mostly insensitive to the used level of theory. The high-level correction is determined in this work through single-point energy calculations on the MEP structure using a spin splitting approach: the CCSD­(T)/aug-cc-pVTZ energy of the triplet state is corrected for the spin pairing energy computed at the CASPT2/aug-cc-pVTZ level. The routines for implementing this correction are integrated within the EStokTP software, which interfaces with electronic structure programs such as MOLPRO or Gaussian. Once the correction potential is determined, the information necessary to define the dividing surfaces for VRC-TST calculations is specified by defining the pivot points for each fragment and their displacement vectors, which are then provided as input to VaReCoF. These calculations are generally split into two parts: short-range and long-range. In long-range calculations, pivot points are typically positioned at the centers of mass of the two fragments. In contrast, for short-range calculations, the pivot points are often fixed at the reactive centers to accurately capture the reactive flux. The output of VaReCoF consists of energy-resolved microcanonical reactive fluxes, which can subsequently be used to determine phenomenological rate constants using a proper master equation solver, such as MESS.^52^ This final step is automated within the EStoKTP framework. To account for recrossing effects, a dynamical correction factor of 0.85 for all reactions except for CH_3_ + H, for which 0.9 is used, is included in the final rate constant, in accordance with previous studies.^17,53^

The strategy used in this work to integrate the ANN with VRC-TST is implemented within VaReCoF, where the optimized ANN model is explicitly invoked in place of the electronic structure software. Given that this function is called thousands of times, minimizing the Python overhead from library imports is crucial. This is accomplished by utilizing the PyTorch’s C++ interface, LibTorch,^54^ which enables the model to be exported as C++ source code. The resulting code can then be compiled into an executable, significantly enhancing the computational efficiency. The DFT calculations are performed with Gaussian,^55^ whereas CCSD­(T) and CASPT2 energies are determined with MOLPRO.^56^

### 2.2 Data Generation and Reference Systems

The training process of the ANN requires generating a specific amount of data, over which the optimization process of the model takes place. Each entry of the data set comprises the six transitional coordinates associated with a specific geometrical orientation of the two fragments, and the relative SPE. The coordinates constitute the input features of the ANN, and their domain is RTS ∈ [2.0, 10.0] Å, AABS1 ∈ [0.0, 180.0] Deg., BABS1 ∈ [-180.0, 180.0] Deg., AABS2 ∈ [0.0, 180.0] Deg., BABS2 ∈ [-180.0, 180.0] Deg., BABS3 ∈ [-180.0, 180.0] Deg. The SPE, in kcal/mol, represents the target and is computed at the CASPT2/cc-pVDZ level of theory employing MOLPRO. The computation of training points is decoupled from the VRC-TST process, enabling optimization of the strategy for performing the thousands of SPE calculations required. Configuration generation is performed using Sobol sequences,^57^ a quasi-random sampling method that efficiently explores multidimensional spaces compared to purely random generation. This approach ensures that configurations are predetermined, and the corresponding SPE calculations can be executed independently, making the process embarrassingly parallel. The active space for these calculations varies depending on the specific reaction but is minimized to reduce human intervention during the computation of large data sets. While multireference calculations can introduce convergence errors, outliers can be addressed during postprocessing by applying energy value checks and removing them from the data set. Figure 3 shows a typical data set produced with Sobol sequences for the system CH_3_ + CH_3_ and RTS going from 2.3 to 4.6 Å.

**Figure 3:**
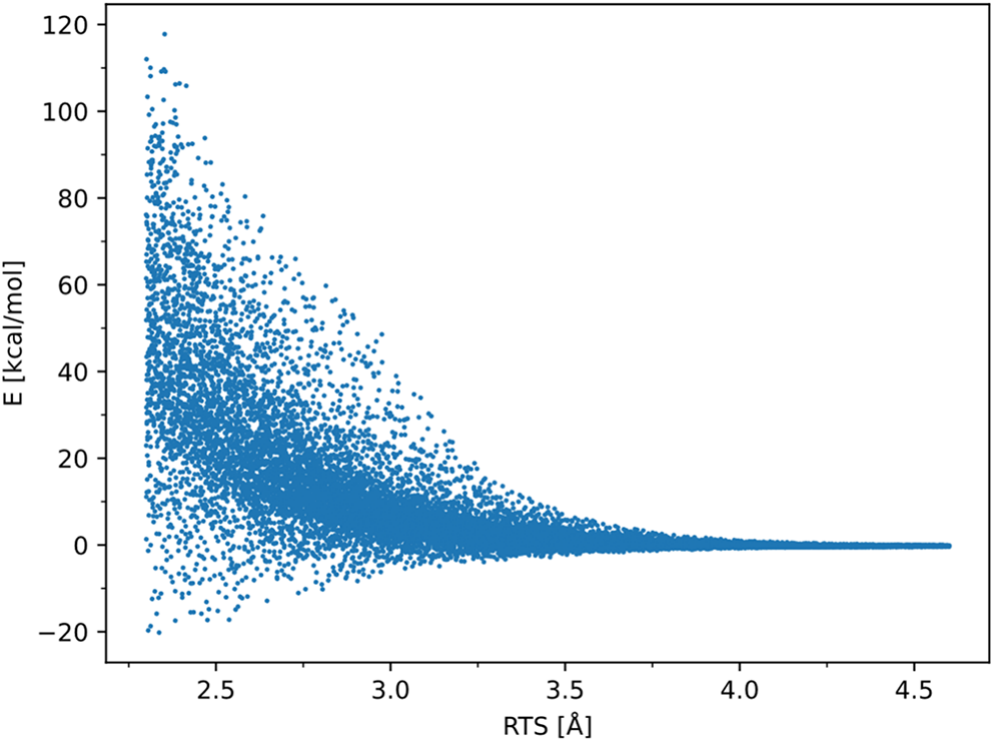
Typical data set produced with Sobol sequences, showing the behavior of the target column and the energy, with respect to the RTS variable.

When dealing with two complex nonlinear fragments, it is common to undersample the region of the phase space with the highest weight in a rate constant computation, where the weight is assigned with a Boltzmann-like function, that is composed of attractive configurations at small interfragment distances, as these configurations present a much smaller probability of being sampled, compared to repulsive configurations. To overcome this hindrance, we embedded into our data set generation algorithm a refinement step. After the first Sobol sequence data generation, a portion, even if small, of optimal configurations is sampled. Starting from such points, a small geometric variation is applied, and additional configurations are generated: this process ensures that the new training points have an energy similar to the starting ones, thus allowing oversampling of the region of interest. It is important to notice that this step is embarrassingly parallel as well since the new set of configurations is generated prior to any calculation, and the time required by the generation is small.

Two additional issues are evident from a typical data set as the one sketched in Figure 3: high-energy configurations may negatively impact the training process by skewing the model, and their contribution to the rate constant is minimal due to their small Boltzmann factor; the PES contains a region dominated by long-range interactions, determined by different electrostatic and polarization forces, as well as dipole and dispersion interactions (characterized by near-zero energy) and a short-range region governed by multireference effects from covalent bond interactions (showing high energy variance). To improve the ANN training, these issues need to be addressed. Configurations with energies exceeding a system-dependent threshold (typically 20–30 kcal/mol) are removed, although some high-energy points are retained to ensure the network learns repulsive behavior. The second issue can be dealt with by dividing the data set based on the RTS variable and training two different models in the two regions, allowing their internal parameters to be optimized for different physical interactions. A range separation distance of 3.5 Å is used to divide the short-range RTS ∈ [2.0, 3.5] Å from the long-range RTS ∈ [3.5, 10.0] Å. For each reaction that we studied in this work, a range separation value of 3.5 Å and an energy threshold around 20–30 kcal/mol proved effective, allowing us to reach the desired accuracy of the ANN potential. Even if these two features are, in general, system-dependent, we provide a successfully tested set of values to be employed. Finding optimal values of the energy threshold and the range separation between the models may be the object of further optimization.

### 2.3 ANN Models

The artificial neural network software is developed in Python, exploiting the PyTorch library^54^ to build, train, and deploy models. The goal of the ANN is to model the nonlinear, multidimensional PESs. The feature space consists of transitional coordinates, requiring the network to have six input neurons corresponding to one distance (measured in Ångström), two angles (measured in degrees), and three dihedrals (also in degrees). The network outputs a single value: the energy (measured in kcal/mol). For cases with fewer DOF, the input layer size is kept fixed, and the missing inputs are set to zero vectors to maintain consistency. Deep neural networks (DNN) based on Rectified Linear Unit (ReLU) activations approximate smooth functions better than shallow neural networks,^58^ since the “curse of dimensionality” is lessened to some extent,^59^ which is particularly important for functions with a large number of independent variables. The use of different activation functions (e.g., polynomial, rather than linear, rectified units^60^) can be shown to improve the approximation properties of DNNs. If the origin of data is known, accuracy and trainability of the ANN can be further enhanced by using application-specific, physics-inspired, activation functions and exploiting known symmetries, asymptotics, and periodicity properties of the target functions.^61^ This is the reason two distinct approaches are adopted and compared: (1) constructing a fully black-box model and (2) developing a physics-inspired model. The black-box model is optimized by systematically increasing the number of hidden layers (HL) and neurons (N) per layer until further improvements in learning, as measured by the root-mean-square error (RMSE), are no longer observed. For instance, starting with a single HL containing 30 N, the architecture was incrementally expanded. The optimal configuration was determined to have two HL, each with 256 N, resulting in a total neuron count of 6 × 256 × 256 × 1. This architecture employs Exponential Linear Unit (ELU) activation functions. To enhance numerical stability during training, a batch normalization^62^ layer is incorporated between the two HLs. To further improve the model’s predictive performance, an additional hidden layer, termed the interaction layer (IL), is introduced between the input layer and the first HL. The IL prioritizes the variable RTS, which holds the greatest physical significance, by allocating a higher number of connections to it compared to a standard fully connected layer. This design, as illustrated in Figure 4(a), ensures that RTS has a greater influence on the model compared to the angular variables. The final black-box architecture includes two batch normalization layers for enhanced stability and predictive accuracy. The physics-inspired neural network (PHINN) adopts a fundamentally different approach, comprising two key components: the periodic block (PB) and the Morse block (MB), as depicted in Figure 4(b).

**Figure 4:**
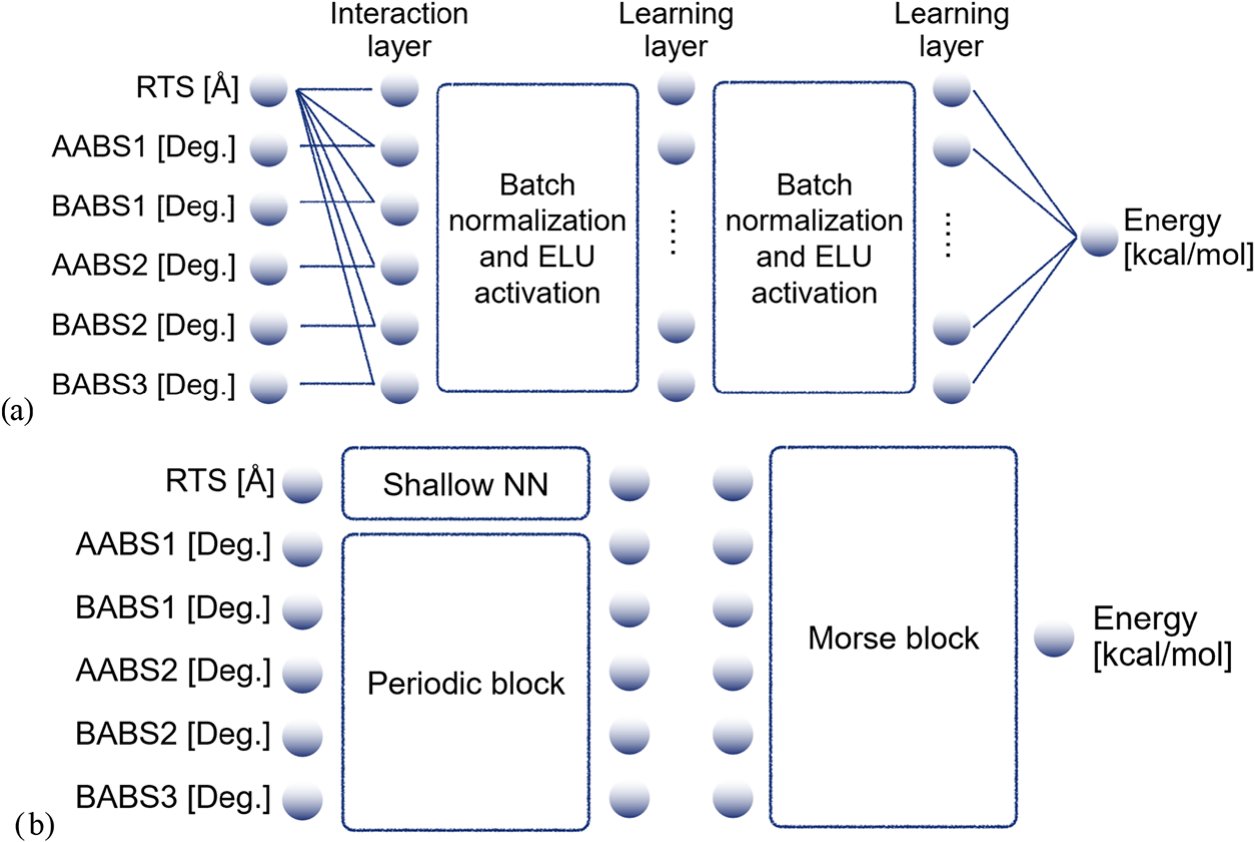
Black-box neural network architecture (a) and the physics-inspired one (b).

The PB is inspired by the work of Dong et al.,^61^ who developed a neural network layer to enforce periodic boundary conditions in numerical solutions of partial differential equations. This concept is adapted here to enforce the periodicity of the PES with respect to the five angular inputs. The PB is based on lemma 2.1,^61^ which deals with function composition involving periodic functions; in particular, given an input layer *x* and an output layer *u*(*x*) on which we want to enforce *C* ^∞^ periodicity with period L, an additional hidden layer is inserted based on periodic functions *v_i_* (*x*) with adjustable parameters. The hyperparameters of this periodic layer are the number of output nodes n and the size m of the set of independent periodic functions *v_i_* (*x*). For fixed m and n, the operations inside the periodic layer are defined as

1 $$v_{i}\left(x\right)=\sigma \left(A_{i}\;\cos\left(\omega x+\phi_{i}\right)+c_{i}\right),1\leq i\leq m$$

2 $$q_{j}\left(x\right)=\sigma \left(\sum_{i=1}^{m}v_{i}\left(x\right)W_{\mathrm{ij}}+B_{j}\right),1\leq j\leq n$$

where σ is the nonlinear activation function, ω = 2π/L. The set of training parameters is composed by *A*_*i*_, ϕ_*i*_, *c*_*i*_, *W*_*ij*_, and *B*_*j*_. We implemented this layer as a user-defined pytorch layer. While *q*_*j*_ (*x*) represents a 1-D periodic layer, the formulation extends naturally to multidimensional cases. A hyperbolic tangent activation function is used. The hyperparameters for the PB are chosen following suggestions by Dong et al.,^61^ selecting *m* = 11, *n* = 30, and an additional linear layer is added at the end of the PB to ensure a 6-D output, that is then fed to the MB. The MB is designed to reproduce a Morse potential relationship between its input and output

3 $$y\left(x\right)=D{\left(1-\exp\left(-A\left(\mathrm{Mx}-R_{e}\right)\right)\right)}^{2}$$

The above equation is implemented through a series of linear and nonlinear operations as follows:

- *y*_1_(*x*) = *Mx* – *R*_e_: linear layer with bias.
- *y*_2_(*x*) = −A*y*_1_(*x*): linear layer without bias.
- *y*_3_(*x*) = (1 – e^*y*_2_(*x*)^)^2^: custom activation function.
- *y*_4_(*x*) = D*y*_3_(*x*): linear layer without bias.

To ensure numerical stability, a normalization layer is included between *y*_2_(*x*) and *y*_3_(*x*), preventing exploding gradients during training due to the exponential activation function. The input layer of the MB has 6 neurons to be consistent with the output of the PB. In this study, the linear layers of the MB feature 32 neurons each, but the dimensions of the latent space in the MB constitute a tunable parameter of the architecture. The term “physics-inspired”, denoting the name of our second model, is specifically used to highlight a difference with respect to physics-informed neural networks that usually incorporate physical laws into the loss function, thus constraining the optimization of the model to adhere to the physics of the system.

### 2.4 Training Scheme

The full data set is partitioned into training, validation, and testing sets. The validation set is employed to tune the network hyperparameters, comprising the learning rate, the number of epochs, and the L2 regularization factor, as well as to monitor overfitting. The test set instead is necessary to assess the model quality after training, measured with the root-mean-square error (RMSE) between the ANN predictions and the multireference calculations used as target. The Adam optimizer is used in the training loop, with betas = 0.8, 0.9.^63^ During training, the target column is standardized, removing its mean and dividing by its standard deviation to ensure any kind of intrinsic bias of the data will not be learned by the network. As introduced in Section 2.2, two different ANNs are trained on a short-range data set with RTS comprised between 2.0 and 3.5 Å, and on a long-range data set with RTS belonging between 3.5 and 10.0 Å. Henceforth, the short-range model will be called *R*_1_, and the long-range one is called *R*_2_. Different values of RMSE are deemed as optimal in the two regions, due to their different energy variance: after numerous experiments, we inferred that a good model for *R*_1_ presents an RMSE below 2 kcal/mol, whereas in the long-range zone, the RMSE must be below 0.5 kcal/mol. Partitioning the training domain into two regions and independently training separate models can result in inconsistencies when predicting potential energy near the separation value. To address these issues and create a unified model, a transfer learning approach is implemented. The optimized short-range *R*_1_ and long-range *R*_2_ models are combined into a third model, *R*_3_, expressed as

4 $$R_{3}\left(x,\theta \right)=s\left(x\right)R_{1}\left(x,\theta \right)+\left(1-s\left(x\right)\right)R_{2}\left(x,\theta \right)$$

where *x* represents the RTS variable, θ is the five angular coordinates, and *s*(*x*) is the joining function defined as follows:

5 $$s\left(x\right)=\{\begin{array}{l} 1\\ a+\mathrm{bx}+cx^{2}+dx^{3}\\ 0 \end{array},\begin{array}{l} if\;x<x_{0},\\ if\;x_{0}\leq x\leq x_{1}\\ if\;x>x_{1} \end{array}$$

The cubic polynomial ensures a smooth transition between the *R*_1_ and *R*_2_. The coefficients *a*, *b*, *c*, and *d* are determined by the following continuity and smoothness conditions

6 $$s\left(x_{0}\right)=1,s\left(x_{1}\right)=0,s^{\prime }\left(x_{0}\right)=0,s^{\prime }\left(x_{1}\right)=0$$

where *x*_1_ and *x*_2_ define the boundaries of the joining region, that in our case are *x*_0_ = 3.0 Å and *x*_1_ = 4.0 Å. This procedure is generalizable, allowing flexibility in varying the separation value or the size of the joining region. The overall workflow for training is summarized in Figure 5.

**Figure 5:**
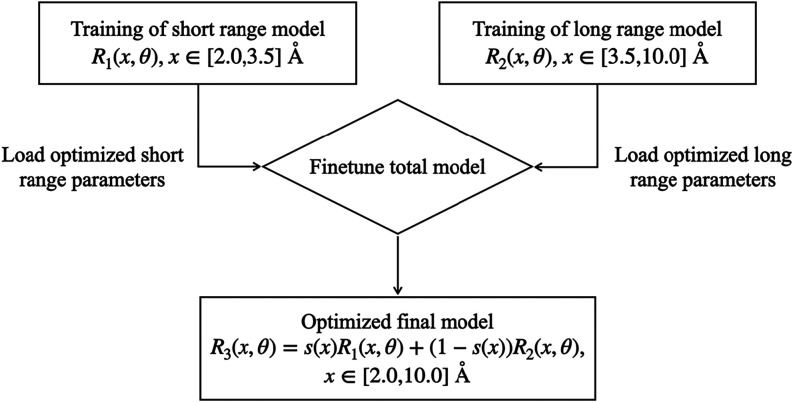
Workflow chart of the different training steps of the model.

The third and last training step consists of transferring the knowledge of the two already optimized models *R*_1_ and *R*_2_ into the global model *R*_3_. Therefore, *R*_3_ is initialized with the pretrained parameters of *R*_1_ and *R*_2_ in their respective regions. The combined model is then fine-tuned without freezing any parameter but choosing a learning rate between 10^–7^ and 10^–9^. Thanks to the properties of *s*(*x*), this fine-tuning primarily affects the joining region while leaving the rest of the training domain nearly unchanged. The result is a single, continuous model across the entire range of RTS, providing robust and optimized predictions. Importantly, the data set used for this final training step is simply the combination of the short-range and long-range data sets, thus requiring no additional data points.

## 3 Results and Discussion

### 3.1 Validation and Comparison of Black-Box and Physics-Inspired Models

#### 3.1.1 Black-Box ANN

The well-established recombination of CH_3_ + CH_3_ serves as an ideal candidate for evaluating the methodology. Initial efforts focused on the black-box architecture without dividing the RTS domain, which spans [2.3, 4.6] Å in this case. Starting with a large training data set of approximately 40,000 points, the model achieved an RMSE below 2.0 kcal/mol. Notably, these error levels were maintained when reducing the training data set to 20,000 points. At this stage, the model was tested in a VRC-TST simulation against a reference calculation to evaluate how these errors influenced the microcanonical reactive fluxes. The results, shown in Figure 6, compare the ANN-based VRC-TST simulation with a reference calculation performed using MOLPRO for Monte Carlo sampling at the CASPT2/cc-pVDZ level of theory using a (2e,2o) active space, employing identical dividing surfaces and pivot points. Across the temperature range of [300, 3500] K, the percentage error in the flux remains below 20%, which was deemed acceptable. Based on these findings, the optimal training data set size was set at 20,000 points, as reducing it further resulted in diminished performance.

**Figure 6:**
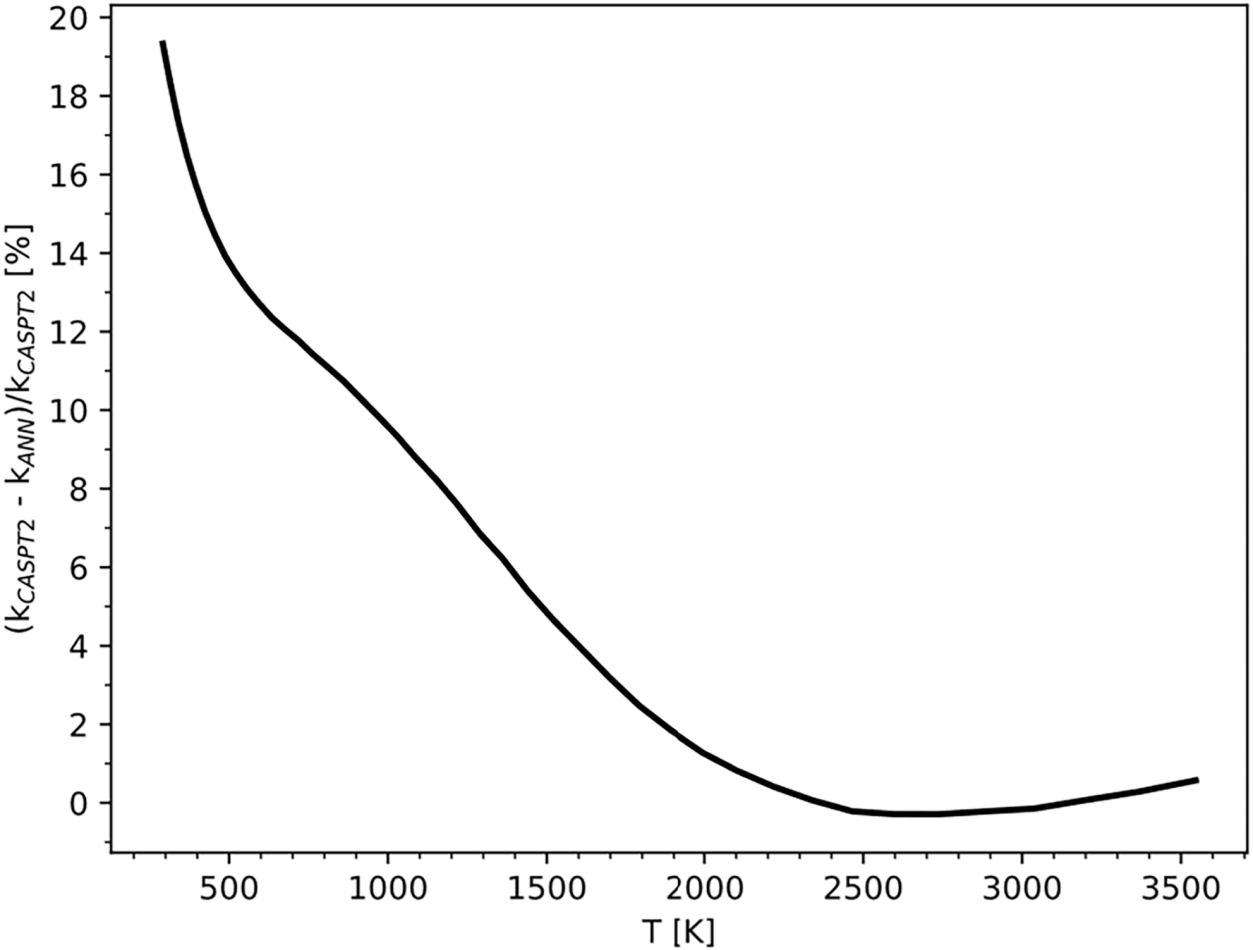
Relative error on high-pressure rate constants calculated as a function of the temperature using the BB network with respect to rate constants obtained using VRC-TST simulations performed by using the standard approach.

#### 3.1.2 Physics-Inspired ANN

Building on the results from the previous section, the PHINN was trained using 20000 training points to compare its performance against the black-box model, obtaining a RMSE of 1.84 kcal/mol. The outcomes, presented in Figure 7, show that the error decreases to below 12.5% over a broader temperature range, from [300, 3500] K for the BB network to [10, 3500] K for the PHINN.

**Figure 7:**
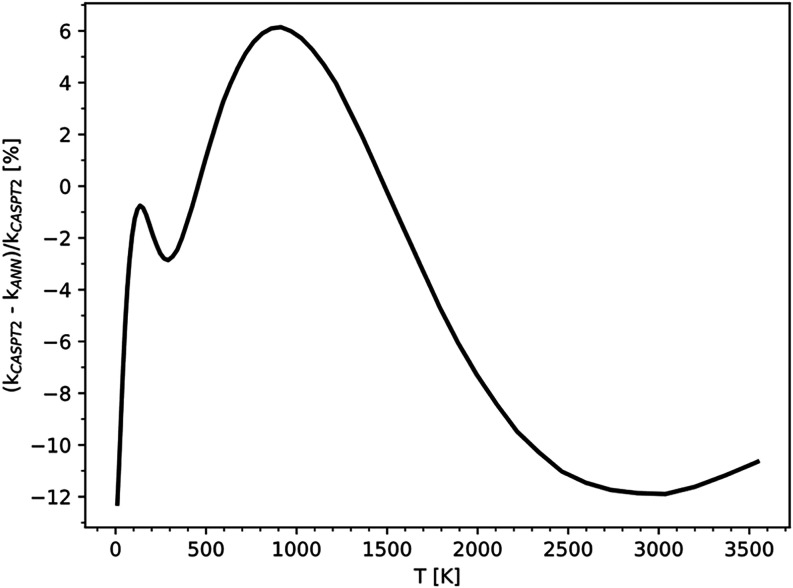
Relative error on high-pressure rate constants, as a function of the temperature, obtained with the PHINN network with respect to rate constants obtained using VRC-TST simulations performed with the standard approach.

The PHINN demonstrates superior predictive power compared to the BB model, delivering high-quality predictions even at very low temperatures and describing much better the long-range features of the potential. However, despite the use of 20,000 training points, the reduction in the number of SPE calculations required for rate constant computation remains limited. To further reduce the number of training points, we addressed the two issues previously outlined in Section 2.3. Following the approach used by Chen et al.,^47^ the data set was split into two regions: short-range and long-range. Separate training phases were conducted, resulting in two distinct models. We achieved an RMSE of 1.86 kcal/mol for the short-range employing 2870 training points and of 0.48 kcal/mol for the long-range with 971 training points. It is clear that this division significantly benefits PES prediction, as the errors remain comparable to the model shown in Figure 7, but the number of training points is reduced by an order of magnitude. After this stage, the two models were combined using the transfer learning approach described in Section 2.4, resulting in a single model then used for VRC-TST simulations. Figure 8 shows the comparison between the PHINN-derived rate constant and the reference high-pressure rate constant. The shaded area highlights that the error between the neural network predictions and the expected results remains below 10%.

**Figure 8:**
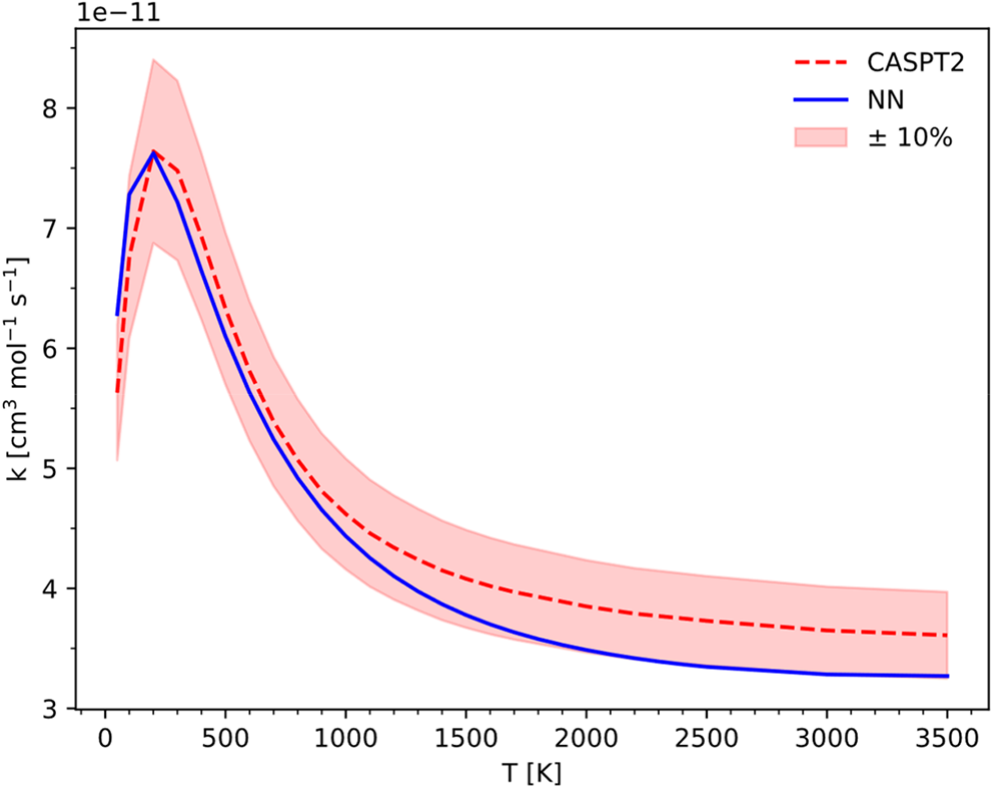
Comparison of the high-pressure limit rate constant obtained with VRC-TST fluxes determined using the standard approach (in dashed red) with respect to the fluxes computed using the PHINN model (in solid blue).

Table 1 compares our findings with those of Chen et al.,^47^ whose electronic structure reference calculations approach differed from ours only for the adopted basis set, cc-pVTZ vs cc-pVDZ, and notably for the architecture of the adopted ANN. As it can be observed the PHINN model achieves a maximum relative error that is smaller by a factor of 2 for this specific reaction, extending the temperature range up to [50, 3500] K, compared to [180, 1200] K.

**Table 1:** Comparison of Relevant Properties of the HPL Rate Constant of CH_3_ + CH_3_ Calculated in This Work with the Work of Chen et al^47^

	this work	Chen et al.
*T* [K]	50–3500	180–1200
max. rel. err.	10.0%	20.0%
lev. theory	CASPT2(2e,2o)/cc-pVDZ	CASPT2(2e,2o)/cc-pVTZ
num. train. P.	3841	2176

### 3.2 Validation of the Methodology

The methodology developed for the CH_3_ + CH_3_ reaction is validated by applying it to four additional reference systems, each representing different complexities in terms of the number of degrees of freedom and the size of the molecular fragments involved. Specifically, the reactions NH_2_ + OH and CH_3_ + H were selected due to their relatively smaller input space, with only five and three DOF, respectively, to assess the model quality in the whole spectrum of possible degrees of freedom. On the other hand, the reactions C_2_H_5_ + CH_3_ and CH_2_CHO + C_2_H_5_ were chosen to test the methodology applicability to systems involving a higher number of heavy atoms, thereby adding complexity and presenting additional challenges in terms of computational accuracy and efficiency. The approach followed for each of these systems remains consistent and involves a series of well-defined steps: first, a data set is generated using Sobol sequences, which are employed for efficient sampling of the multidimensional space. The data set is then split into short-range and long-range regions, and separate models are trained for each region. The number of training points used is reported in Table 2.

**Table 2:** Total Number of Training Points Used for Each Reaction, Divided by the Short- and Long-Range Region

reaction	short range	long range	total
CH_3_ + CH_3_	2871	971	3841
NH_2_ + OH	2317	653	2970
CH_3_ + H	2800	300	3100
C_2_H_5_ + CH_3_	2673	953	3626
CH_2_CHO + C_2_H_5_	2285	883	3168

Afterward, the two models are fine-tuned and combined to form a global model. This combined model is then used to perform a VRC-TST simulation, and the resulting high-pressure rate constant is compared to the reference values for validation. Interestingly, optimal model performances are obtained using similar hyperparameters of the PHINN for all systems: a learning rate of 10^–3^, batch sizes of 256 and 64 for short and long ranges, respectively, and 2000 as number of epochs. The comparison between rate constants computed using the PHINN model, those obtained through standard VRC-TST calculations, and literature values is shown in Figure 9 for all of the investigated systems.

**Figure 9:**
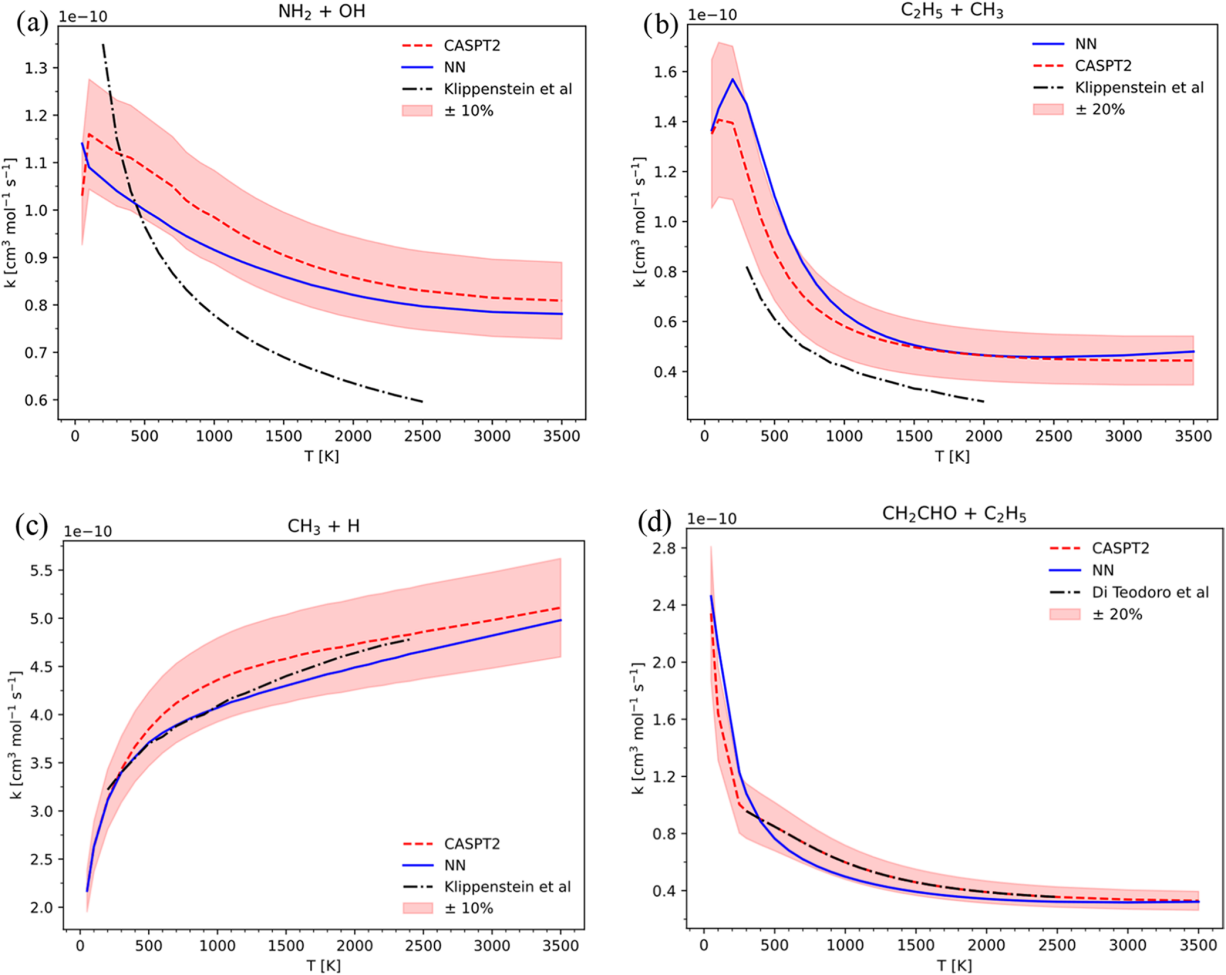
Comparison of HPL rate constants obtained with VRC-TST fluxes computed with standard VRC-TST calculations (dashed red) and with the PHINN (solid blue), for the recombination of NH_2_ + OH in (a), compared with Klippenstein et al.,^64^ of C_2_H_5_ + CH_3_ in (b), compared with Klippenstein et al.,^17^ of CH_3_ + H in (c), compared with Klippenstein et al.,^53^ and of CH_2_CHO + C_2_H_5_ in (d), compared with Di Teodoro et al.^27^

From these results, we observe that for systems containing up to two heavy atoms, the relative error in the predicted rate constant remains below 10%. However, as the size of the molecular fragments increases, the error tends to rise, reaching 20% for systems with more complex fragment structures. It is interesting to report the number of random samples used in the VRC-TST simulations employing both methods, using the same setup for integral convergence, i.e., maximum number of samples per dividing surface equal to 5000 and 5% of standard deviation of the calculated flux. Using the PHINN, the VRC-TST procedure required 26270 samples for C_2_H_5_ + CH_3_, 51985 samples for CH_2_CHO + C_2_H_5_, 25320 samples for CH_3_ + CH_3_, 9665 samples for CH_3_ + H, and 12140 samples for NH_2_ + OH. These numbers should be compared with the ones of Figure 1, which we report here for clarity: 21330 for C_2_H_5_ + CH_3_, 46020 for CH_2_CHO + C_2_H_5_, 24800 for CH_3_ + CH_3_, 10190 for CH_3_ + H, and 12955 for NH_2_ + OH. The similarity between the number of random samples suggests a similar level of orientational integral convergence in the VRC-TST procedure, which thus does not influence the discrepancy between the two calculated rate constants evidenced in Figures 8 and 9. Using a significantly smaller training data set demonstrates that the number of SPE calculations required by this methodology to compute a rate constant within the VRC-TST framework is reduced by nearly a factor of 4, a similar result was also found by Chen et al.^47^ This efficiency is evident from the data reported in Figure 1, where the smallest system examined in this study, CH_3_ + H, necessitates over 10,000 SPE evaluations with traditional approaches. Extending this comparison to the other systems investigated further emphasizes the advantages of the proposed methodology. Beyond the reduction in SPE calculations, PHINN also offers a substantial speedup in the evaluation of configuration energies. The trained network can produce an energy value in milliseconds, a process that is independent of the level of theory, basis set, or fragment size. Measuring the time required by the VRC-TST simulation employing the ANN, we observed a time reduction of 1 order of magnitude in the CH_3_ + CH_3_ case, limited by our interface between VaReCoF and the neural network potential. The calculated rate constants in Figure 9 are also compared to theoretical calculations available in the literature,^17,27,53,64^ showing a reasonable agreement. The present work does not aim at reproducing rate constants approaching experimental accuracy; it is instead focused on reproducing selected theoretical results employing the NN methodology. The discrepancies highlighted in Figure 9(a,b) are thus not alarming. However, the NN potential can be used to set up a VRC-TST simulation with an increased number of dividing surfaces and pivot point displacements, thus providing a more accurate description of the bottleneck in the reactive flux. Concerning the system C_2_H_5_ + CH_3_, Figure 10 illustrates that the updated VRC-TST calculation employing the NN potential produces a rate constant approximately 10% lower than the original value, thereby improving agreement with literature results.^17^ This enhanced simulation utilized 328565 NN SPEs, a quantity typically unfeasible for multireference electronic structure methods applied to a system of this size.

**Figure 10:**
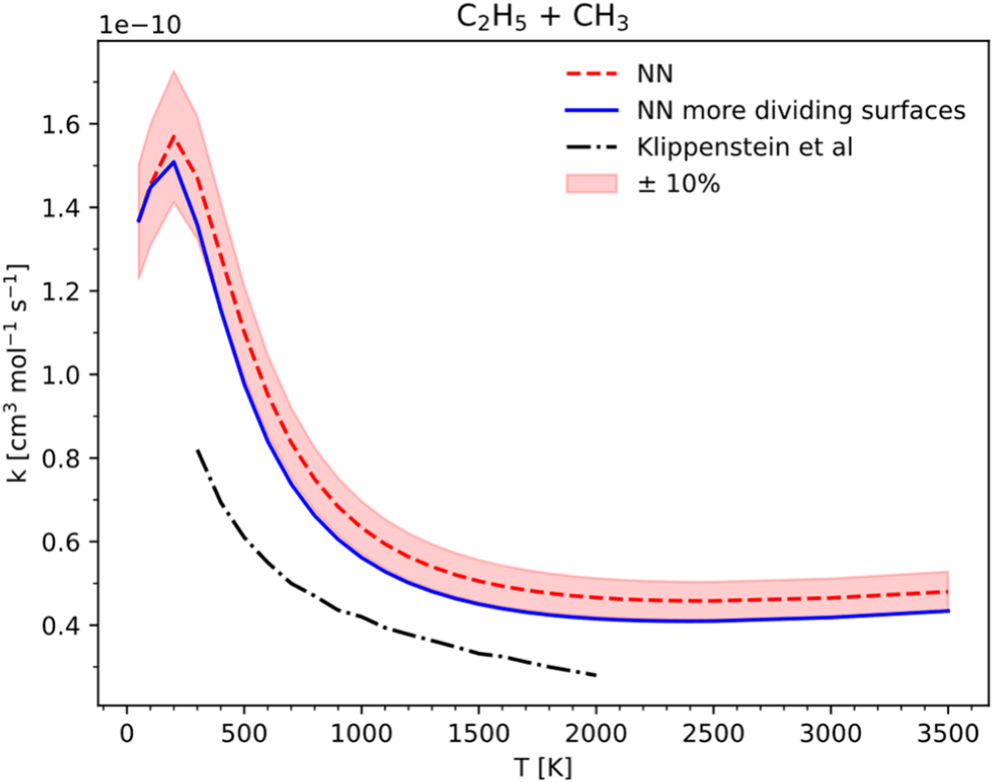
HPL rate constants for C_2_H_5_ + CH_3_ showing the original calculation with the NN, in red, and the calculation with the enlarged grid of dividing surfaces and pivot point displacements, in blue, compared to literature results.^17^ The shaded area indicates a 10% difference from the original NN calculation.

Analyzing Figure 9(a,b,d), a specific trend is observable: the error of the NN rate constant with respect to the standard VRC-TST calculations decreases when the temperature increases. As the temperature increases, the minimum of the reactive flux moves toward shorter interfragment separations. The error decrease may be due to different concurring effects, such as the greater number of sampling points used in the short-range region, which leads to better generalization capabilities of the model at high temperature, or to the increase in the interaction energy, which implies a tighter transition state whose number of states progressively approaches that predicted by the RRHO approximation. However, such a trend seems not to be present in Figure 9(c), for CH_3_ + H, signaling that if the system is simple enough, long-range interactions can be learnt by the model employing an extremely small amount of training points, since just 300 points were used for CH_3_ + H. There is an additional advantage in employing the proposed machinery for computing rate constants. Decoupling the computation of training points from the VRC-TST sampling allows one to check the PES prior to any rate constant calculation. Therefore, the quality of the points can be assessed, removing outliers or convergence errors and making sure only physically insightful configurations are included in the computation. A practical example of the aforementioned issue is provided by the reaction CH_2_CHO + C_2_H_5_. When employing a (4e,4o) active space, the system is very prone to converge to an incorrect active space configuration, particularly at large interfragment separations, unless a specific strategy is used to compute the SPE. This phenomenon is depicted in Figure 11. In panel (a), results obtained using a Hartree–Fock guess directly opening the active space to (4e,4o) are shown, whereas in panel (b), a specific rationale is adopted, consisting of multiple sequential wave function guesses, with a gradual expansion of the active space. While in panel (b) one can appreciate the energy of the system converging to an asymptote, in panel (a) one can spot three distinct bands of data for interfragment distances greater than 8 Å, indicative of incorrect convergence to the proper active space in CASPT2 calculations.

**Figure 11:**
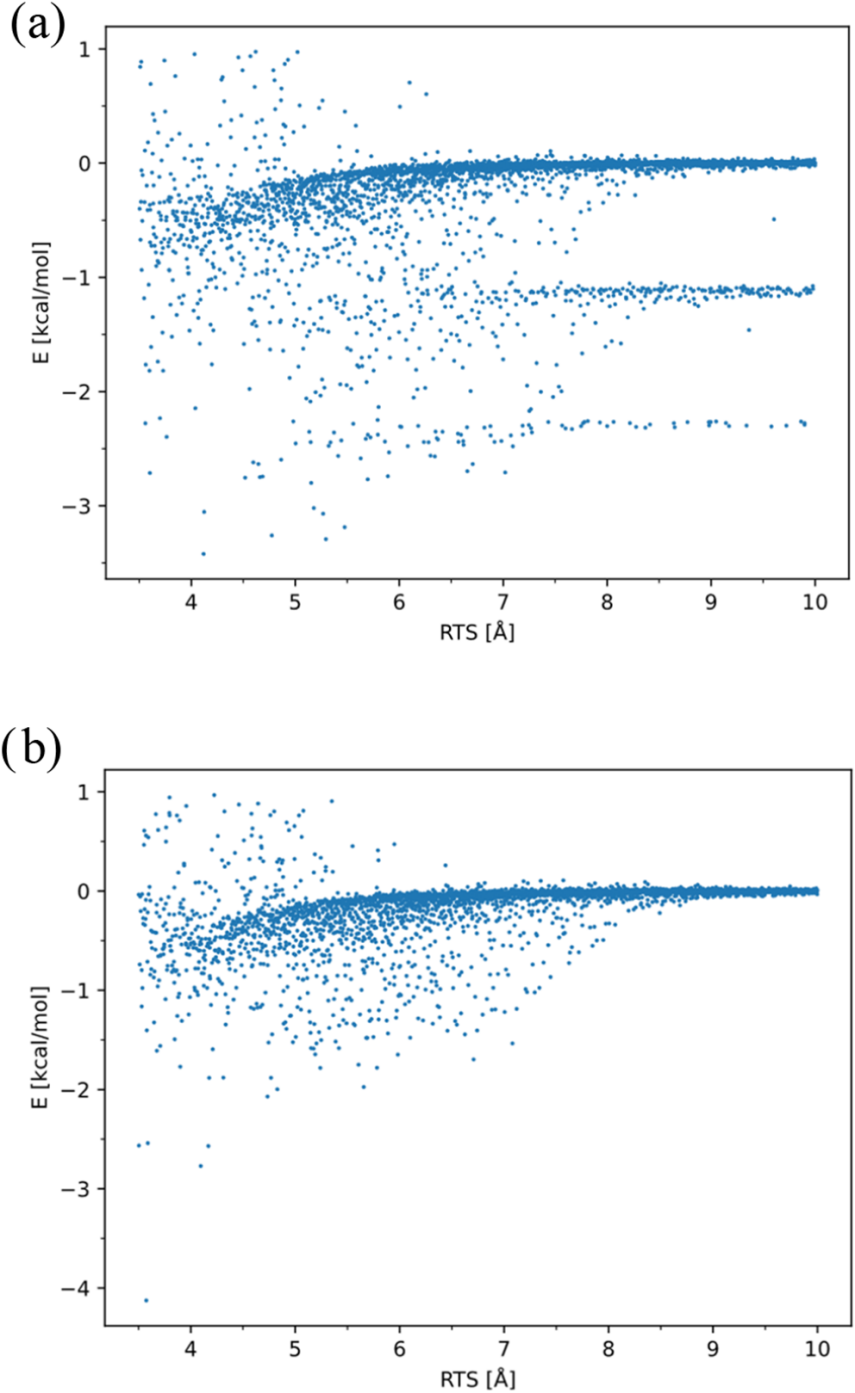
Data set for the system CH_2_CHO + C_2_H_5_ with RTS going from 3.5 to 10.0 Å computed: (a) From an unrestricted Hartree–Fock guess directly opening the active space to (4e,4o), and (b) using as a guess a wave function generated for a specific geometry.

## 4 Conclusions

This work addresses the computational challenges arising in the rate constant computation of gas-phase barrierless reactions. These reactions, which lack a distinct energy barrier along the reaction coordinate, present unique difficulties due to the need to model large-amplitude, anharmonic motions, and the multireference nature of the reacting configurations in proximity of the transition state. Such difficulties are overcome within the VRC-TST framework that entails a Monte Carlo sampling of the PES, mapped as a function of the relative degrees of freedom, mapped into the large-amplitude motions of the two frozen interacting fragments participating in the chemical reaction. This procedure generally requires tens of thousands of SPE evaluations with complex multireference electronic structure calculations. To mitigate this bottleneck, we propose to use an ANN to fit the multidimensional scalar PES. In order to develop a computationally efficient and robust procedure much work has been devoted to the data set production stage, that exploiting Sobol sequences resulted in an embarrassingly parallel task through which the necessary SPEs can be evaluated in principle through a single job submission stage. Developing both black-box and physics-inspired ANN models, it was shown that the latter one has superior predictive capabilities at parity of data set’s size. For both ANN, each entry corresponds to the six geometrical degrees of freedom and the configuration energy. Two specific techniques were applied to the data to shrink the training set as much as possible. The first step was the filtration of high-energy configurations, setting up a system-dependent energy threshold in the 20–30 kcal/mol range for the systems investigated in this work. The second one consisted of dividing the data set into two regions based on the intermolecular distance, as the ANN must describe interactions of different physical nature: long distances (long-range) are governed by many different electrostatic and polarization forces as well as dipole and dispersion interactions, whereas short distances (short-range) are dominated by the multireference character of the forming covalent bond. The latter refinement implied training two different models for the short- and long-range. To avoid inconsistencies near the separation value, which was set to 3.5 Å, a transfer learning approach was adopted consisting of loading the pretrained parameters of the two previously optimized models into a third unified model, built with a cubic polynomial joining function whose coefficients were determined imposing continuity and smoothness boundary conditions on the joining region. The transfer learning-derived high-pressure rate constants are in optimal agreement with the theoretical reference calculations: the relative error is below 10% for the systems CH_3_ + CH_3_, NH_2_ + OH, CH_3_ + H and below 20% for C_2_H_5_ + CH_3_, CH_2_CHO + C_2_H_5_, demonstrating the robustness and the quality of the ANN predictions of the PES. It is also shown that employing the NN, enhancing the precision of a VRC-TST calculation by increasing the number of dividing surfaces is computationally feasible and leads to an improved agreement with literature results. The overall number of SPE needed to compute a VRC-TST rate constant for a gas-phase barrierless reaction was thus substantially reduced by about 1 order of magnitude. Additional time savings emerge from the negligible time required by the ANN to produce an energy value for a given configuration compared to what is needed by electronic structure software. Most importantly, the prediction time and the number of parameters of the ANN developed do not depend on the level of theory, basis set size, and fragment size. In conclusion, the integration of Artificial Neural Networks with VRC-TST has proven to be a highly effective and promising approach for addressing the computational challenges associated with calculating rate constants for barrierless reactions. The methodology proposed in this work led to substantial gains in computational efficiency without sacrificing the accuracy required for reliable rate constant predictions. Further directions of this work entail the extensive usage of this methodology on specific reaction classes as well as the development of potentials to be used in frozen fragment trajectory simulations. Also, we believe that the methodology developed here, thanks to the possibility it gives of introducing a systematic error tracking procedure and, given the efficient use of computational time, to perform calculations at a level of theory sufficiently high that would make high-level corrections to SPEs superfluous, will help considerably in the development of black-box automatic procedures for the estimation of rate constants through VRC-TST.
